# Diosmetin exerts anti-oxidative, anti-inflammatory and anti-apoptotic effects to protect against endotoxin-induced acute hepatic failure in mice

**DOI:** 10.18632/oncotarget.15413

**Published:** 2017-02-16

**Authors:** You Yang, Xiao-Bao Gong, Li-Gua Huang, Zhen-Xu Wang, Rong-Zhen Wan, Peng Zhang, Qing-Yan Zhang, Zhu Chen, Bao-Shun Zhang

**Affiliations:** ^1^ College of Animal Science and Technology, Southwest University, Chongqing, P. R. China; ^2^ College of Pharmaceutical Sciences, Southwest University, Chongqing, P. R. China; ^3^ Chongqing Institute for Food and Drug Control, Chongqing, P. R. China; ^4^ The Ninth People’s Hospital of Chongqing, Chongqing, P. R. China

**Keywords:** diosmetin, acute hepatic failure, oxidative stress, inflammatory, apoptosis, Pathology Section

## Abstract

To investigate the effects and mechanism of diosmetin on acute hepatic failure (AHF), an AHF murine model was established through administration of lipopolysaccharides/D-galactosamine (LPS/D-GalN). *In vitro*, diosmetin scavenged free radicals. *In vivo*, diosmetin decreased mortality among mice, blocked the development of histopathological changes and hepatic damage, and suppressed levels of inflammatory mediators and cytokines. In addition, diosmetin prevented the expression of phosphorylated IKK, IκBα, and NF-κB p65 in the NF-κB signaling pathway, and JNK and p38 in the MAPK signaling pathway. Diosmetin also inhibited hepatocyte apoptosis. Thus, diosmetin exerts protective effects against endotoxin-induced acute hepatic failure in mice. The underlying mechanisms are antioxidation, NF-κB signaling inhibition, inflammatory mediator/cytokine attenuation, and hepatocyte apoptosis suppression. Diosmetin is thus a potential drug candidate for use in the treatment of acute hepatic failure.

## INTRODUCTION

Acute hepatic failure (AHF) is defined as the sudden clinical syndrome of severe hepatocellular dysfunction accompanied by hepatic encephalopathy in a healthy human [1, 2]. The loss of hepatocellular function leads to metabolic derangements, including clotting factor synthesis, gluconeogenesis and ureogenesis, impairment of plasma detoxification, neurologic complications, and ultimately, multiorgan failure [3]. AHF has a variety of etiologies, such as viral hepatitis [4], excessive alcohol [5], and drug-induced hepatotoxicity [6]. When patients experience life-threatening liver failure, no effective therapy is available apart from liver transplantation [7]. Liver transplantation is not widely used because of the shortage of donor livers and the expense of transplantation. Some researchers have considered cell-based therapies for liver failure because these therapies result in fewer traumas, are relatively simple to apply, cost less, and are reversible and repeatable [8, 9]. Unfortunately, cell-based therapies are not appropriate for most patients. Therefore, a continuing search for a promising hepatoprotective agent is necessary.

Nuclear factor kappa B (NF-κB) is important for liver physiology and function [10] because it can be activated by many different stimuli and it maintains tissue homeostasis, controls disease development, promotes cell survival and activates innate and adaptive immune responses. The expression of several genes involved in the inflammatory response, including inducible nitic oxide synthase (iNOS), cyclooxygenase-2 (COX-2), and tumor necrosis factor-α (TNF-α), is activated at the transcriptional level by the inducible transcription factor NF-κB [11]. The NF-κB signaling pathway is a factor in several liver diseases, including hepatitis, cirrhosis, and hepatocellular carcinoma. Furthermore, the NF-κB signaling pathway is a potential target for hepatoprotective agents such as antioxidants and nuclear factor kappa-B kinase (IKK) inhibitors. These drugs disrupt activity at different levels and treat liver disease [12].

**Figure 1:**
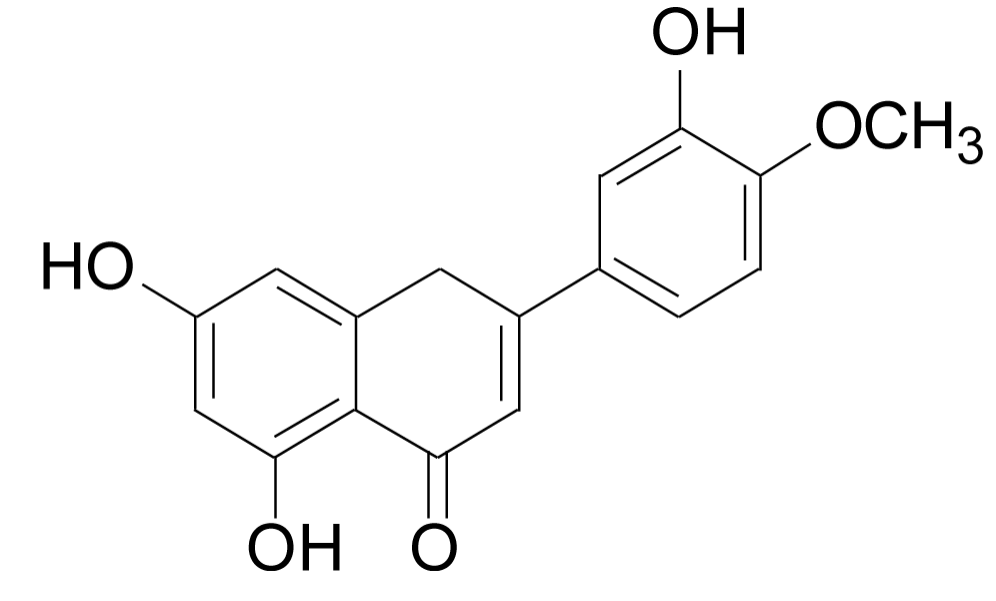
The chemical structure of diosmetin (DIOS)

Diosmetin (DIOS [3´, 5, 7-trihydroxy-4´-methoxyflavone]) (Figure 1) is a bioflavonoid that is abundant in citrus fruits [13], legume leaves, and spermin. DIOS exerts antioxidant [14], anti-inflammatory [15–18], antiapoptotic [19], antimutagenic [20], and antibacterial [21] effects. DIOS promotes strong cellular antioxidant activity in human monocytes by preventing the generation of intracellular ROS and the formation of malondialdehyde (MDA), and by increasing the effects of the intracellular antioxidant enzymes superoxide dismutase (SOD), catalase (CAT), and glutathione peroxidase (GPx) [14]. DIOS exerts an anti-inflammatory effect by reducing NO production and TNF-α release induced by lipopolysaccharides in murine microglia and macrophages [15]. DIOS suppresses mouse-ear edema induced by 12-O-tetradecanoyl- phorbol-13-acetate (TPA) [16] and decreases the enzyme activities of p38α mitogen-activated-protein kinase (p38α) and c-Jun-N-terminal kinase 3 (JNK3) [17]. In addition, DIOS suppresses apoptosis in T48 cells by the activation of protein kinase B (AKT) and protein kinase B (ERK) phosphorylation [19]. However, little information about the protective effect of DIOS on LPS-induced acute hepatic failure is available. Therefore, this study was performed to determine its interaction and potential mechanism in a murine model.

## RESULTS

### Abilities of DIOS to scavenge free radicals *in vitro*

**Figure 2:**
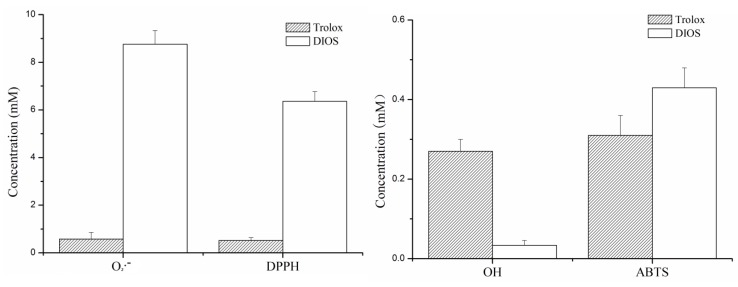
Half maximal inhibitory concentrations (IC _**50**_, mM) of DIOS and Trolox on the four free radicals (O_**2**_·^−^, DPPH, ·OH, and ABTS) ***in vitro***.

Four superoxide free radicals (O_2_·^−^), 2, 2-diphenyl-1-picrylhydrazyl (DPPH), hydroxyl radical (·OH), and 2, 2´-azino-bis-3-ethylbenz-thi-azoline-6-sulphonic acid (ABTS^+^), were inhibited by pretreatment with DIOS (DIOS+LPS/D-GalN), and their IC_50_ values were 8.76, 6.36, 0.034, and 0.43 mM, respectively. The inhibition of ·OH by DIOS pretreatment exhibited an extremely significant difference (*P* < 0.01) compared with the positive control Trolox (IC_50_ = 0.27 mM) (Figure 2).

### Effects of DIOS on LPS/D-GalN-induced mortality and histopathological changes

**Figure 3:**
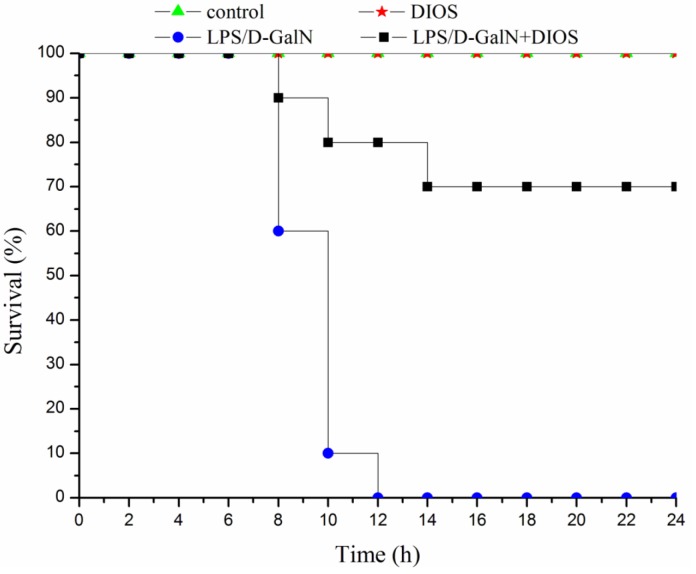
Mortality in DIOS-treated AHF mice induced by LPS/D-GalN endotoxin The mortality of the mice were measured every 2 hours for 24 hours. The administered dosages were as follows: LPS 10 μg/kg, D-GalN 400 mg/kg; DIOS 50 mg/kg +LPS/D-GalN.

**Figure 4:**
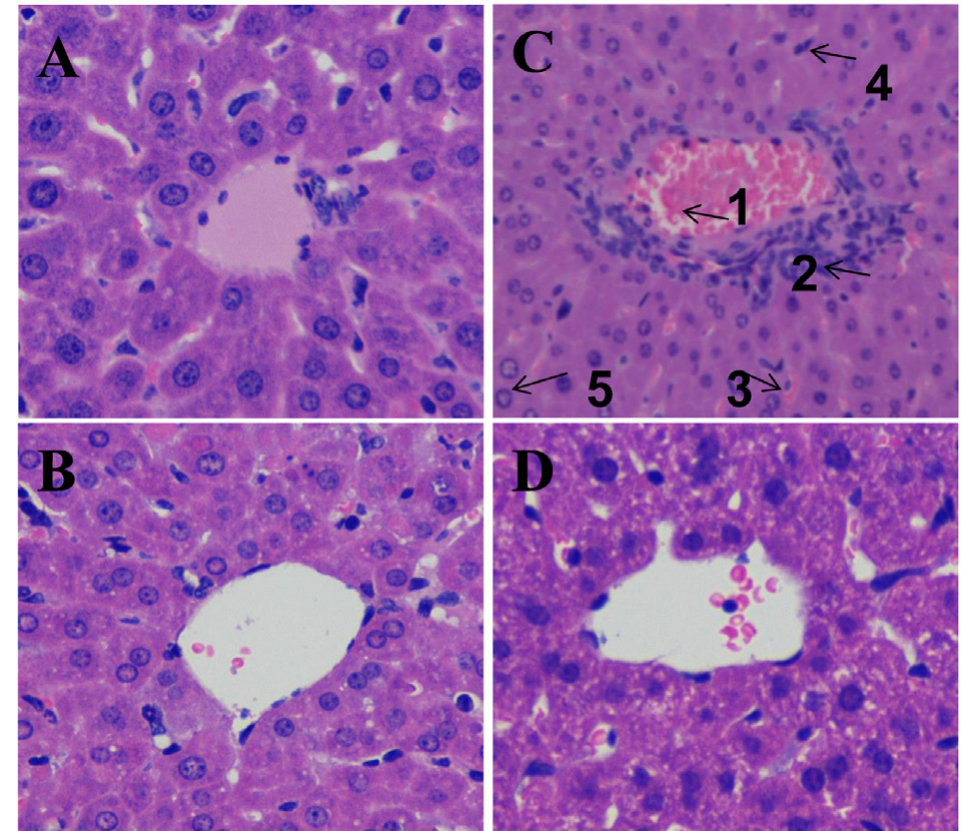
Effect of DIOS on liver tissue of AHF mice after administration of LPS/D-GalN endotoxin Representative histopathological changes in liver obtained from mice in different groups are indicated. **A**. Control, **B**. DIOS, **C**. LPS/D-GalN (LPS 10 μg/kg, D-GalN 400 mg/kg; 1: central venous hyperemia; 2: inflammatory infiltration; 3: hepatic sinusoid hyperemia; 4: hepatocyte necrosis; 5: the loss of hepatic architecture-vacuolation), **D**. DIOS (50 mg/kg) +LPS/D-GalN.

The effect of DIOS pretreatment on LPS/D-GalN-induced mortality in mice is depicted in Figure 3. All mice in the control and DIOS groups survived for 24 hours. However, after administration of LPS/D-GalN, the mice started to die at 8 hours, and up to 100% mortality had occurred at 12 hours. At 24 hours after administration of LPS/D-GalN, mortality only reached 30% in mice pretreated with DIOS. Thus, DIOS+LPS/D-GalN decreased LPS/D-GalN-induced mortality in mice. The effect of DIOS pretreatment on LPS/D-GalN- induced histopathological changes is shown in Figure 4. Histological analysis of the mouse hepatic tissue was performed with H&E staining assay. The hepatic architectures in the control and DIOS groups were normal. Administration of LPS/D-GalN caused significant histological changes, including inflammatory infiltration, hepatocyte necrosis, hemorrhage, and the loss of hepatic architectures-vacuolation. By contrast, pretreatment with DIOS effectively blocked the development of histopathological changes induced by LPS/D-GalN.

### Effects of DIOS on hepatic damage and oxidative and inflammatory markers

**Figure 5:**
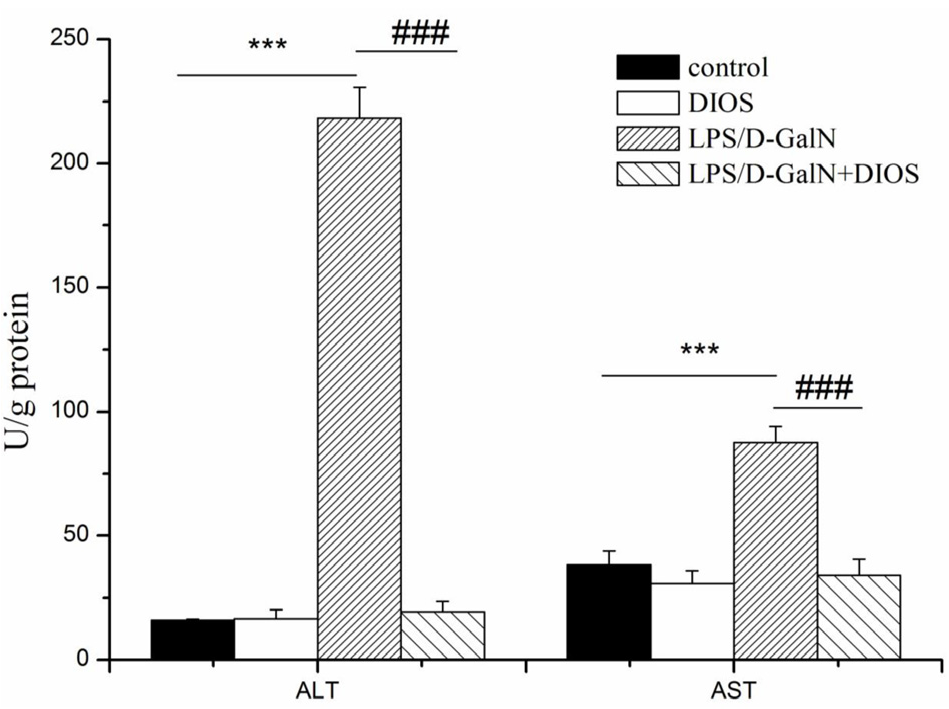
Activities of ALT and AST in serum of mice treated with DIOS after LPS/D-GalN administration All data are presented as means ± SD (*n* = 10). ^**^*P < 0.01 *vs* control group; ^###^*P* < 0.01 *vs* LPS/D-GalN group.

Alanine and aspartate aminotransferase (ALT and AST) activities were measured to evaluate hepatic damage (Figure 5). Administration of LPS/D-GalN significantly increased ALT and AST. Pretreatment with DIOS decreased ALT and AST and resulted in a significant difference (DIOS+LPS/D-GalN *vs* LPS/D-GalN, *P* < 0.01 and *P* < 0.05, respectively). DIOS pretreatment attenuated the hepatic damage induced by LPS/D-GalN.

**Figure 6:**
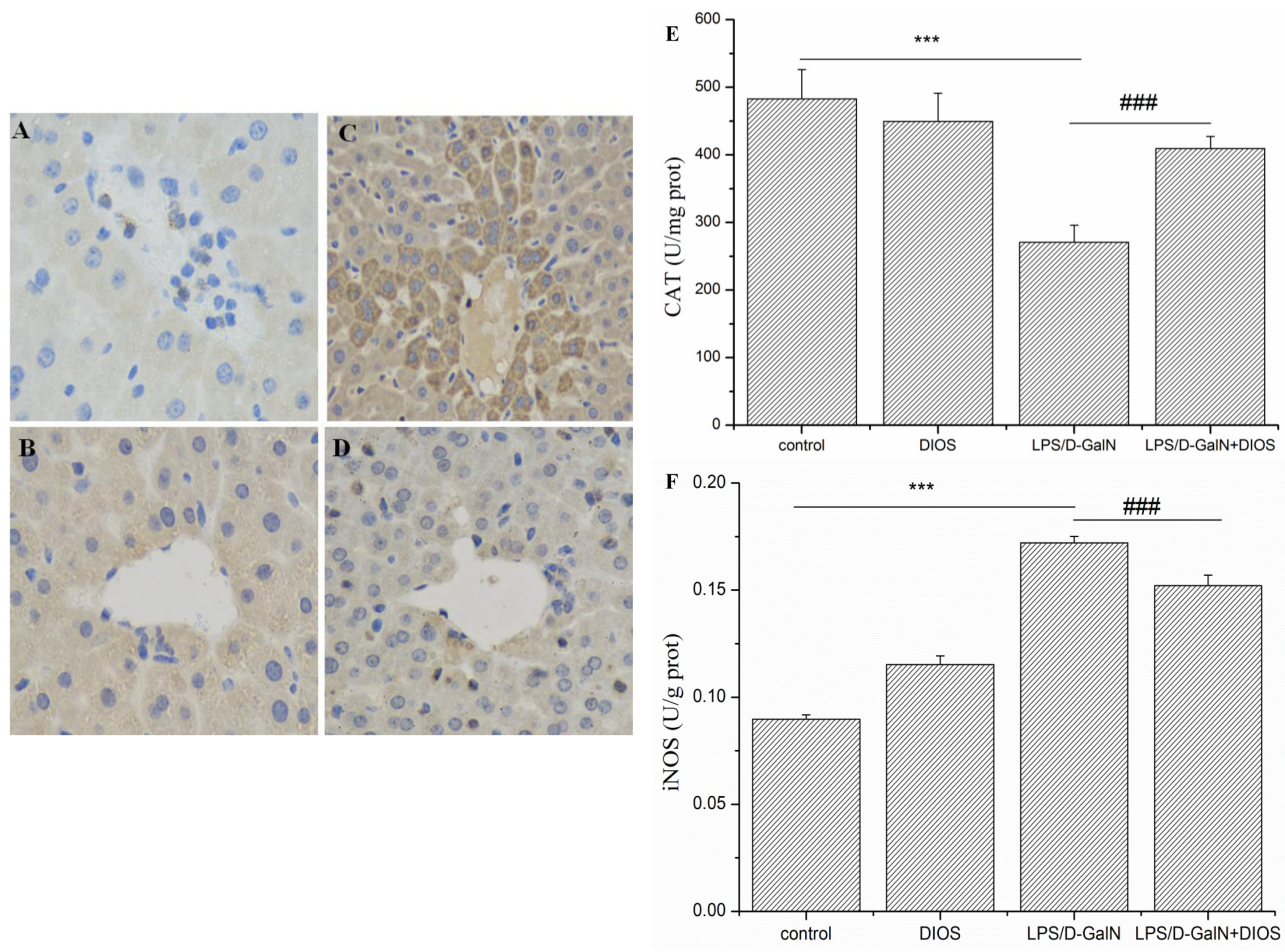
Activities of CAT and iNOS in serum of mice treated with DIOS after LPS/D-GalN administration **A**. Control, **B**. DIOS, **C**. LPS/D-GalN (LPS 10 μg/kg, D-GalN 400 mg/kg), and **D**. DIOS (50 mg/kg) +LPS/D-GalN are indicated in the representative photomicrographs of iNOS immunoreactivity. Parts **E**. and **F**., respectively, show the activity of CAT and iNOS in different groups. All data are presented as means ± SD (*n* = 10).^**^*P < 0.01 *vs* control group; ^###^*P* < 0.01 *vs* LPS/D-GalN group.

The quantitative analysis of nitic oxide synthase (iNOS), malondialdehyde (MDA), catalase (CAT), superoxide dismutase (SOD), and total antioxidant capacity (T-AOC) serves as the assessment of LPS/D-GalN-induced oxidant damage (Figure 6 and Table 1). The enzyme activities of CAT, SOD, and T-AOC decreased in the LPS/D-GalN group and increased in the DIOS treatment group. However, the levels of iNOS and MDA increased in the LPS/D-GalN group and were suppressed by DIOS+LPS/D-GalN.

**Table 1:** The levels of DIOS on the concentrations of COX-2, PGE2, MDA, T-AOC, and SOD in mice serum after LPS/D-GalN administration (x̅±s, *n* =10)

Group	COX-2 (ng/L)	PGE2 (ng/L)	MDA (nmol/mL)	T-AOC (U/mL)	SOD (U/mL)
Control	61.7 ± 4.7	122.0 ± 7.0	7.3 ± 0.8	4.5 ± 1.0	29.3 ± 5.7
DIOS	60.5 ± 6.2	115.2 ± 4.9	7.9 ± 2.2	4.6 ± 1.1	26.9 ± 5.0
Model	79.4 ± 3.6^**^*	202.1 ± 17.4^**^*	24.7 ± 5.4^**^*	1.9 ± 0.5^**^*	18.6 ± 3.4^**^*
DIOS+LPS/ D-GalN	59.1 ± 3.8 ^###^	148.4 ± 9.6^###^	16.0 ± 2.4^##^	3.8 ± 0.5^###^	27.8 ± 1.9^##^

***P < 0. 01 vs control group; ##*P* < 0.05, ###*P* < 0. 01 vs model group. Model group was injected with LPS/D-GalN (LPS, 10 μg/kg bodyweight; D-GalN, 400 mg/kg body weight, dissolved in saline). DIOS group is administered DIOS (50 mg/kg body weight/day in Tris-buffer) for 6 days. DIOS+LPS/D-GalN group was treated with DIOS (50 mg/kg body weight/day in Tris-buffer) for 6 continuous days, and then injected with LPS/D-GalN endotoxin.

**Table 2:** The levels of DIOS on the concentrations of COX-2, PGE2, MDA, T-AOC and SOD in mice liver after LPS/D-GalN administration (x̅±s, *n* =10)

Group	COX-2 (ng/L)	PGE2 (ng/L)	MDA (nmol/mL)	T-AOC (U/mL)	SOD (U/mL)
Control	52.5 ± 3.9	143.5 ± 7.6	3.2 ± 0.4	0.4 ± 0.0	39.3 ± 6.6
DIOS	53.9 ± 5.0	131.2 ± 12.6	3.8 ± 0.6	0.3 ± 0.1	31.5 ± 6.5
Model	70.7 ± 2.5^**^*	166.8 ± 11.4^*^	5.4 ± 0.6^**^*	0.1 ± 0.1^**^*	14.8 ± 4.1^**^*
DIOS+LPS/ D-GalN	59.4 ± 4.5 ^##^	141.7 ± 10.6 ^##^	4.0 ± 0.7 ^##^	0.2 ± 0.1 ^##^	24.7 ± 4.6 ^##^

**P < 0. 05, ***P < 0. 01 vs control group; ^##^*P* < 0.05 vs model group. Model group was injected with LPS/D-GalN (LPS, 10 μg/kg bodyweight; D-GalN, 400 mg/kg body weight, dissolved in saline). DIOS group is administered DIOS (50 mg/kg body weight/day in Tris-buffer) for 6 days. DIOS+LPS/D-GalN group was treated with DIOS (50 mg/kg body weight/day in Tris-buffer) for 6 continuous days, and then injected with LPS/D-GalN endotoxin.

Prostaglandin E_2_ (PGE_2_) and COX-2 are critical accelerators of pathogenesis and have emerged as therapeutic targets in inflammatory diseases. The enzyme activities of COX-2 and PGE_2_ are determined in Table 1. After the administration of LPS/D-GalN, the two activities increased, whereas they were reduced by the DIOS treatment.

### Effects of DIOS on the NF-κB signaling pathway

**Figure 7:**
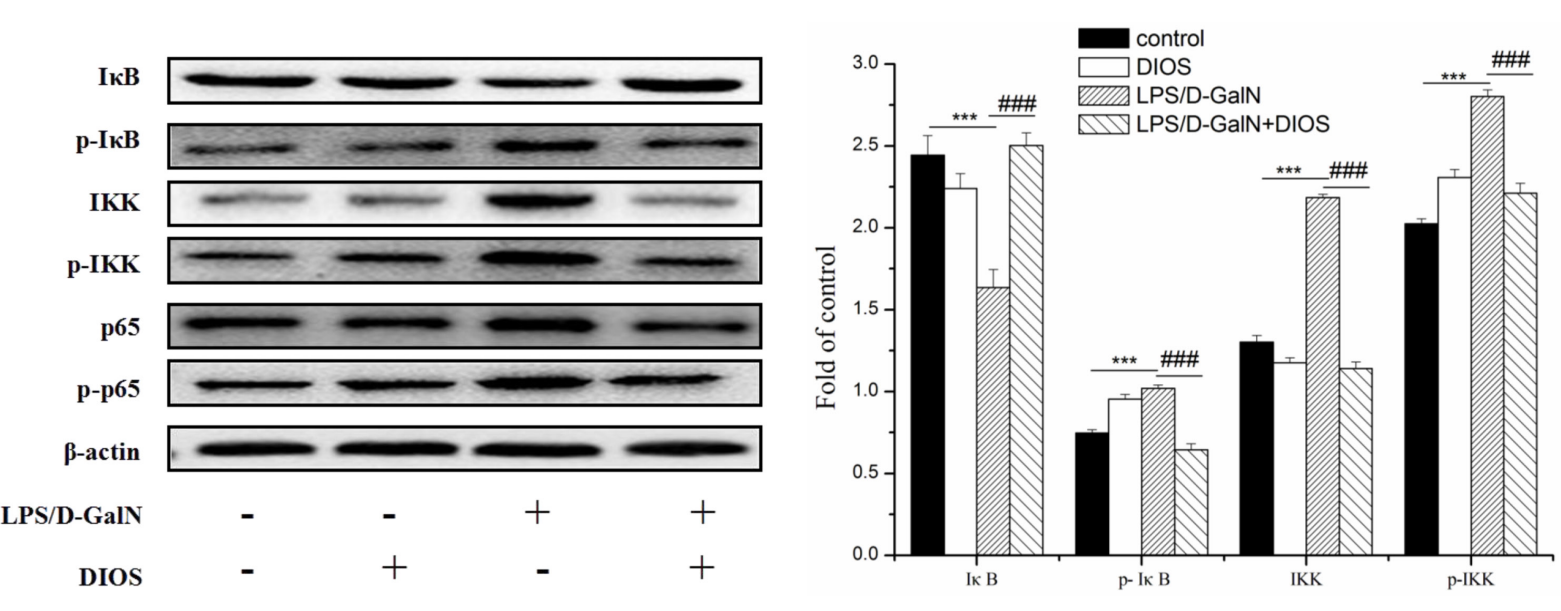
Effect of DIOS on the NF-KB signaling pathway after administration of LPS/D-GalN endotoxin All data are presented as means ± SD (*n* = 10). ^**^*P < 0.01 *vs* control group; ^###^*P* < 0.01 *vs* LPS/D-GalN group.

**Figure 8:**
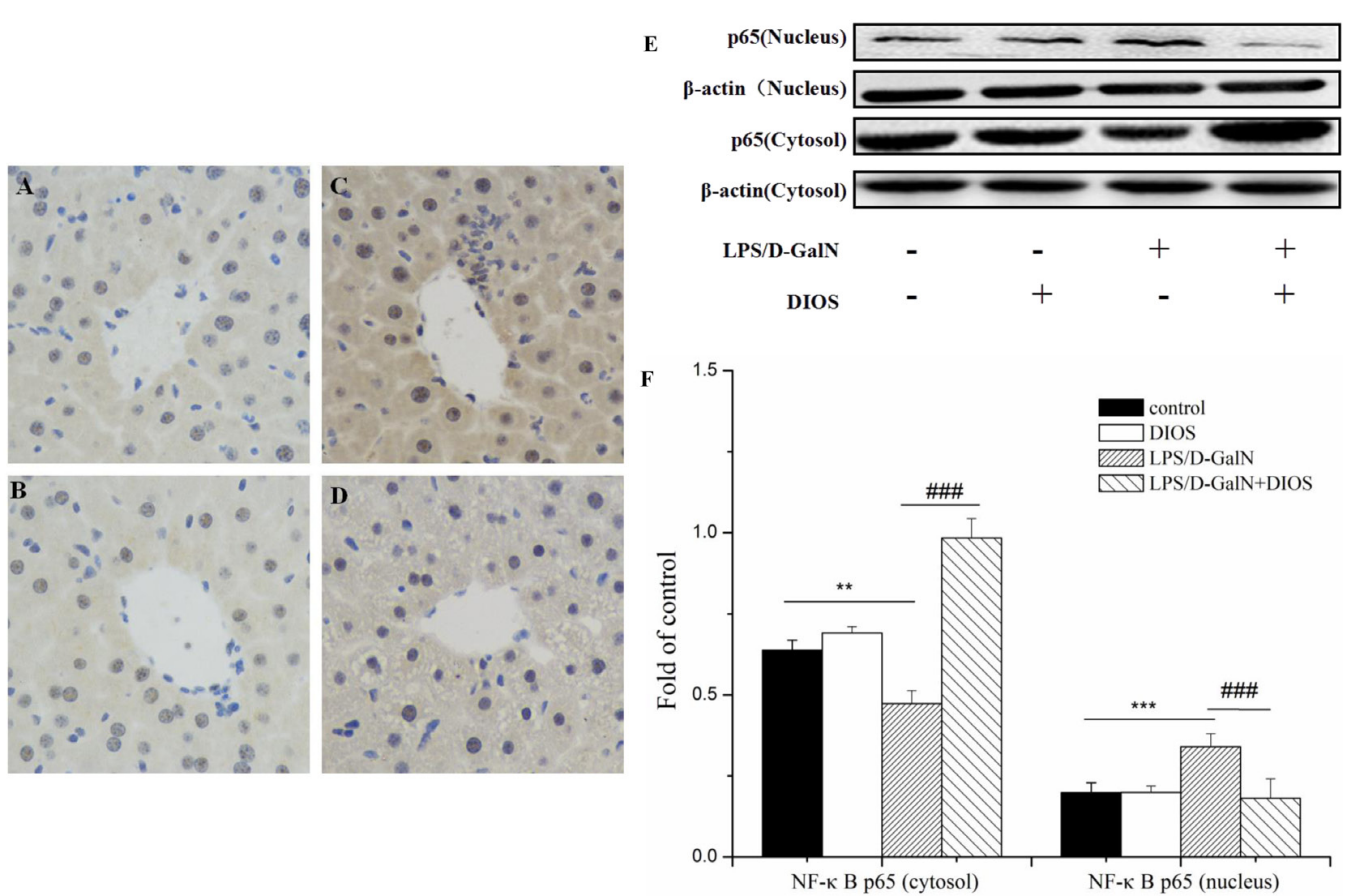
Effect of DIOS on the expression and translocation of NF-κB(p65) after LPS/D-GalN administration **A**. Control, **B**. DIOS, **C**. LPS/D-GalN (LPS 10 μg/kg, D-GalN 400 mg/kg), and **D**. DIOS (50 mg/kg)+LPS/D-GalN are indicated in the representative photomicrographs of NF-κB (p65) immunoreactivity. **E**. and **F**. Cytosol and nucleus NF-κB (p65) expressions were determined by western blot analysis. All data are presented as means ± SD (*n* = 10). ^*^*P < 0.05, ^**^*P < 0.01 *vs* control group; ^###^*P* < 0.01 *vs* LPS/D-GalN group.

**Figure 9:**
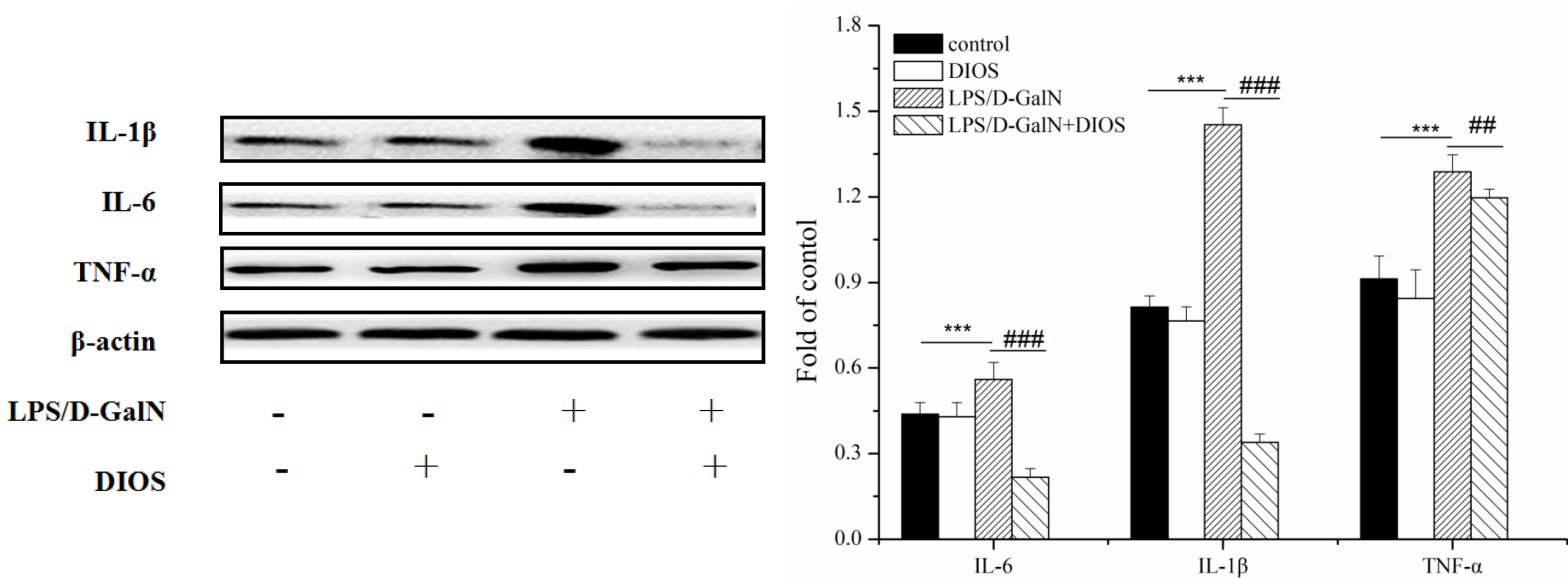
Effect of DIOS on the expressions of IL-1β, IL-6, and TNF-α after LPS/D-GalN administration All data are presented as means ± SD (*n* = 10). ^**^*P < 0.01 *vs* control group; ^###^*P* < 0.01 *vs* LPS/D-GalN group.

Inflammatory mediators/cytokines cause the pathogenesis of acute hepatic failure. To investigate the effect of DIOS on the NF-κB signaling pathway, the expressions of the key proteins were examined by use of a western blot assay. Administration of LPS/D-GalN, increased the phosphorylation of IKK and the inhibition of NF-κB alpha (IκBα) and NF-κB (p65 subunit). Nevertheless, pretreatment with DIOS blocked these increases (Figure 7). Although LPS/D-GalN triggered activation of NF-κB (p65), which is the major subunit of NF-κB, the effect of DIOS on the transcription of p65 still needed to be studied. As shown in Figure 8, the expression of p65 in the nucleus was measured more than that in the cytosol of the LPS/D-GalN group. However, the expression of p65 in the nucleus was reduced after treatment with DIOS. Protein levels of proinflammatory cytokines such as tumor necrosis factor-α (TNF-α), interleukin-1β (IL-1β), and interleulin-6 (IL-6), increased in the LPS/D-GalN group and decreased in the DIOS +LPS/D-GalN group (Figure 9). The inhibitory effect of DIOS on acute hepatic failure is perhaps related to the NF-κB signaling pathway.

### Effects of DIOS on the MAPK signaling pathway

**Figure 10:**
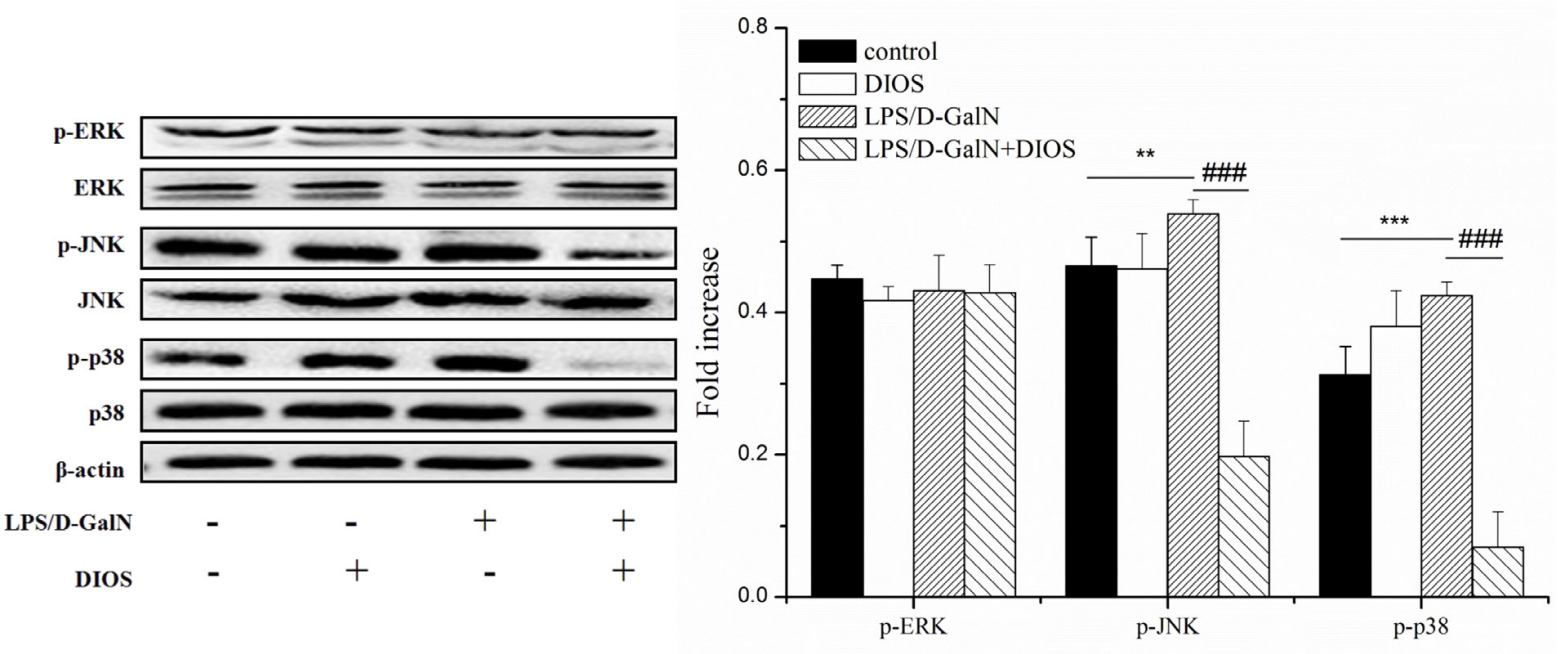
Effect of DIOS on the MARK signaling pathway after administration of LPS/D-GalN endotoxin All data are presented as means ± SD (*n* = 10). ^*^*P < 0.05, ^**^*P < 0.01 *vs* control group; ^###^*P* < 0.01 *vs* LPS/D-GalN group.

The phosphorylation of JNK, ERK, and p38 MAPK proteins were triggered and increased in the LPS/D-GalN group (Figure 10). The phosphorylated levels of JNK and p38 MAPK proteins were reduced in the DIOS+LPS/D-GalN group. The phosphorylation of ERK remained unchanged in the DIOS+LPS/D-GalN group. The result indicated that DIOS inhibited the JNK and p38 MAPK proteins in the MAPK family without affecting ERK. The MAPK signaling pathway might correlate with the DIOS anti-inflammation properties.

### Effects of DIOS on LPS/D-GalN-induced hepatocyte apoptosis

**Figure 11:**
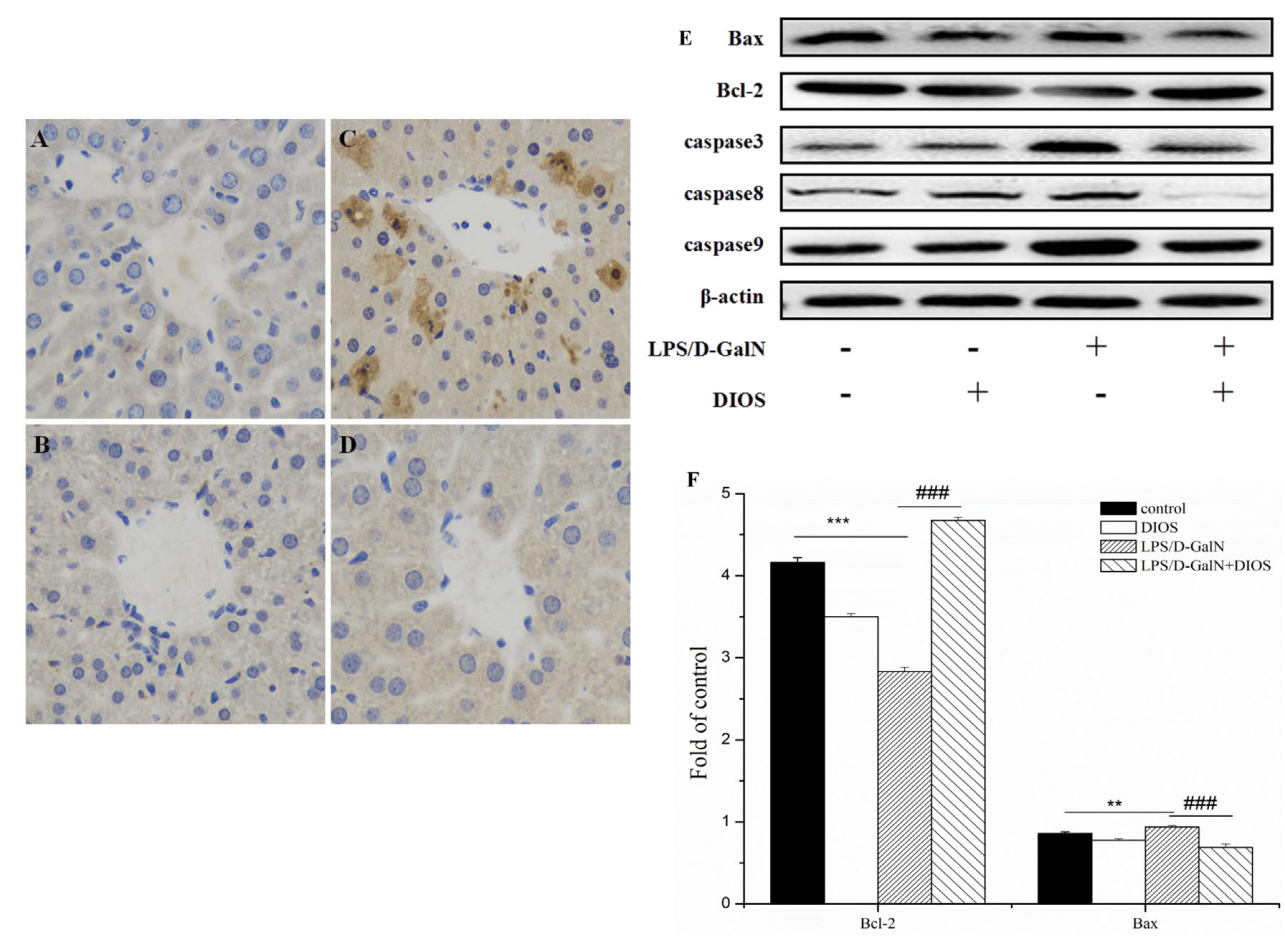

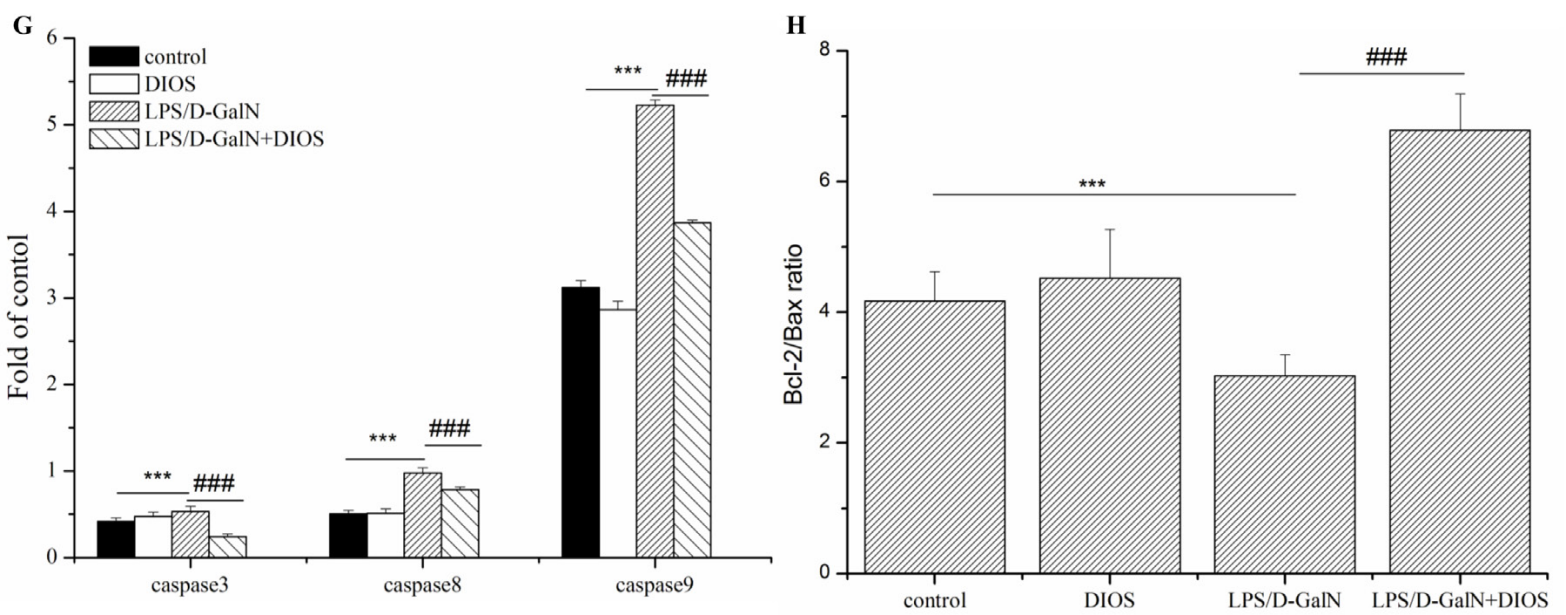
Effect of DIOS on the expressions of apoptotic-related proteins after LPS/D-GalN administration **A**. Control, **B**. DIOS, **C**. LPS/D-GalN (LPS 10 μg/kg, D-GalN 400 mg/kg), and **D**. DIOS (50 mg/kg)+LPS/D-GalN are indicated in the representative photomicrographs of caspases 3 immunoreactivity. **E**., **F**., and **G**. Bax, Bcl-2, and Caspase 3, 8, 9 expressions were examined by western blot analysis. **H**. Bcl-2/Bax ratios were calculated with their protein expressions. All data are presented as means ± SD (*n* = 10). ^*^P < 0.05, ^**^*P < 0.01 *vs* control group; ^###^*P* < 0.01 *vs* LPS/D-GalN group.

To investigate hepatocyte apoptosis induced by LPS/D-GalN, a western blot assay was performed (Figure 11). Bcl-2 associated X (Bax) increased and B-cell lymphoma 2 (Bcl-2) decreased with the administration of LPS/D-GalN, respectively. However, pretreatment with DIOS prevented the expression of Bax and increased the expression of Bcl-2. The Bcl-2/Bax ratio increased in this condition. The expression of caspase 3, 8, and 9 increased with administration of LPS/D-GalN and decreased with administration of DIOS. The result revealed that DIOS inhibited hepatocyte apoptosis induced by LPS/D-GalN.

## DISCUSSION

The liver performs hundreds of critical functions to maintain homeostasis and health. The liver disease AHF severely affects health and is life threatening. Thus, finding a hepatoprotective agent is vital for clinical therapy.

LPS and D-GalN-induced hepatitis is a well-established model of liver injury promoted by macrophages [22]. In this study, an AHF murine model was established successfully with i.p. injection of LPS/D-GalN. Administration of DIOS prevented the high mortality, inflammatory infiltration, hepatocyte necrosis, hemorrhage, and the loss of hepatic architectures induced by LPS/D-GalN. Furthermore, DIOS attenuated histopathological changes and diminished the activities of ALT and AST stimulated by endotoxin.

Oxidative stress reflects the imbalance between the generation of reactive oxygen species (ROS) such as superoxide radicals (O_2_·^−^), singlet oxygen, hydrogen peroxide (H_2_O_2_), and hydroxyl radicals (·OH) and the biochemical mechanisms that detoxify and repair the damage resulting from reactive intermediates [23, 24]. Oxidative stress damages lipids, proteins, and DNA, which results in cell growth arrest, senescence, or death [25], and many disease processes of clinical interest have oxidative stress as an underlying etiology. In the present study, treatment with DIOS inhibited O_2_·^−^, ·OH, DPPH, and ABTS^+^, and DIOS strongly scavenged MDA in mouse serum, which acted as an intracellular radical in the inflammatory response. iNOS has been implicated in both DNA damage induction and aberrant cell signaling in various tissue and cells [26]. Our result demonstrated that the level of iNOS increased with administration of LPS/D-GalN. However, treatment with DIOS suppressed iNOS activity. SOD and CAT are major radical-scavenging antioxidant enzymes in the human body [27]. SOD catalyzes the highly reactive superoxide anion O_2_·^−^ into O_2_ and hydrogen peroxide (H_2_O_2_). CAT converts the H_2_O_2_ generated within cells to H_2_O and O_2_ and prevents the conversion of H_2_O_2_ into a more active species, such as ·OH. Total antioxidant capacity (T-AOC) served as a preventive index of oxidant damage. Our results indicated that administered LPS/D-GalN reduced the activities of SOD and CAT and attenuated T-AOC capacity. Pretreatment with DIOS reduced LPS/D-GalN-induced depression of SOD and CAT.

Prostaglandin E_2_ (PGE_2_) is a potent lipid activator produced by the inducible form of the enzyme COX-2 in inflammatory cells. PGE_2_ and COX-2 are critical accelerators of pathogenesis and therefore have emerged as therapeutic targets in inflammatory diseases [28]. Our results demonstrated that pretreatment with DIOS reduced the enzyme activities of COX-2 and PGE_2_, which were increased significantly by the administration of LPS/D-GalN.

NF-κB is one of the major transcription factors for gene expression in the inflammatory response. The activation of NF-κB is triggered by the phosphorylation of IκB, and then NF-κB is dissociated from the inactive cytoplasmic complex. IκB is phosphorylated by IκB kinases (IKKs), which results in its ubiquitination and proteasomal degradation. Degradation of IκBα results in the release of NF-κB p65 and causes the translocation of activated NF-κB into the nucleus, which then causes the transcription of target genes [29–31]. Along with the NF-κB activation, the levels of several cytokines, such as IL-1β, IL-6, and TNF-α, involved in apoptosis and inflammation, increased. Our findings revealed that pretreatment with DIOS blocked the phosphorylation of IKK, IκBα, and NF-κB p65, reduced the expression of p65 in the nucleus, and decreased the levels of IL-1β, IL-6, and TNF-α. The inhibitory effect of DIOS on AHF induced by LPS/D-GalN is increased by the NF-κB signaling pathway.

ERK, JNK, and p38 MAPK are the main components of the MAPK family. The MAPK pathway has emerged as one of the major factors in the intracellular signaling cascades that are part of the pro-inflammatory response [32, 33]. In this study, we demonstrated that administration of LPS/D-GalN initiated the activation of ERK, JNK, and p38 MAPK. Pretreatment with DIOS blocked the phosphorylated JNK and p38 MAPK. The MAPK signaling pathway might correlate with the DIOS anti-inflammation properties.

The MAPK signaling pathways promote a variety of cellular activities, including proliferation and apoptosis, in response to certain stimuli. The anti-apoptotic protein (Bcl-2) and the pro-apoptotic protein (Bax) are the two types of Bcl-2 family proteins. Bak and some pro-apoptotic proteins stimulate cell apoptosis, but Bcl-2 inhibits apoptosis [34]. The Bcl-2/Bax ratio as an index is more important than either promoter alone in determining apoptosis. Our results indicated that DIOS administration increased the Bcl-2/Bax ratio induced by LPS/D-GalN. After hepatocytes were stimulated by LPS/D-GalN, mitochondrial ultrastructural damage was caused by caspase-mediated apoptosis. Caspase family proteases (caspase 3, 8, and 9) triggered the apoptotic process [35]. Administration of DIOS, reduced the phosphorylated levels of caspase 3, 8, and 9 compared with LPS/D-GalN. The result revealed that DIOS inhibited hepatocyte apoptosis.

We found that pretreatment with DIOS attenuated AHF in mice induced by LPS/D-GalN endotoxin. The underlying mechanism of DIOS inhibition of AHF is suppression of oxidative stress, attenuation of hepatocyte apoptosis, decrease of the production of inflammatory mediator/cytokines, and block of the activation of the NF-κB and MAPK signaling pathways, with restraint of the expression and phosphorylation of the relevant proteins. Therefore, DIOS has potential as a therapy for AHF.

## MATERIALS AND METHODS

### Chemicals

DIOS (purity 99% by HPLC) was provided by Chemistry Institute of Pharmaceutical Resource of Southwest University (China). LPS (*Escherichia coli* 055:B_5_) and D-GalN were purchased from Sigma-Aldrich Co. LLC. (Shanghai, China). Diagnostic kits used for the determination of AST, ALT, CAT, iNOS, PGE_2_, COX-2, MDA, SOD, and T-AOC activities were obtained from the Nanjing Jiancheng Institute of Biotechnology (Nanjing, China). Rabbit IL-6, IL-1β, IκB, p-IκB, IKK, p-IKK, p38, p-p38, JNK, p-JNK, ERK, p-ERK, NF-κB (p65), Bax, Bcl-2, caspase 3, caspase 8, caspase 9, β-actin, mouse TNF-α polyclonal primary antibodies, goat anti-mouse IgG-HRP-conjugated secondary antibody and goat anti-rabbit IgG-HRP-conjugated secondary antibody were purchased from Proteintech (Wuhan, China), and total protein extraction kits were from Sangon Biotech Co. Ltd (Shanghai, China).

### DIOS scavenging of free radicals *in vitro*

DIOS scavenging of the free radicals ·OH, O_2_·^−^, DPPH and ABTS *in vitro* was assessed by comparison with corresponding methods from the literature.

### Animals and treatment

Male and female Kunming mice weighing 20 g (± 2 g) were purchased from the Chongqing Medical University Experimental Animal Center (China), Certificate of Conformity: 0001802. Animal level: SPF; rearing conditions: SPF-class animal laboratory at room temperature 23°C (± 2 °C), relative humidity of 50%, 15 times per hour for the wind. The mice were maintained on tap water and rodent food ad libitum and acclimatized for at least one week before use. Mice were randomly divided into four groups with 10 mice in each group. The control group received normal saline. The DIOS group was administrated DIOS (50 mg/kg body weight/day in Tris-buffer) for 6 days. The LPS/D-GalN group was administered saline once daily for 6 days. One hour after the final saline treatment, the mice were injected with LPS/D-GalN (LPS, 10 μg/kg bodyweight; D-GalN, 400 mg/kg body weight, dissolved in saline). The DIOS+LPS/D-GalN group was treated with DIOS (50 mg/kg body weight/day in Tris-buffer) for 6 continuous days. Six hours after the final DIOS treatment, the mice were injected with LPS/ D-GalN (LPS, 10 μg/kg body weight; D-GalN 400 mg/kg body weight, dissolved in saline). All injections were executed intraperitoneally. Animals were sacrificed 6 hours after LPS/D-GalN administration. Blood samples were collected from the retroorbital venous plexus and centrifuged at 4°Cfor 10 minutes at 1400×g in glass tubes. The serum was stored at −80°C in polystyrene tubes until use. Livers of both groups were harvested immediately and snap-frozen in liquid nitrogen for histopathology, immunohistochemistry, and western blot assay.

### Ethics statement

All the animal experiments in this study were carried out in accordance with the Guide for the Care and Use of Laboratory Animals, formulated by Chongqing Municipal People's Government, and approved by the Experimental Animal Management Committee of Chongqing, China.

### Determination of lethality and histopathological changes

Survival rates of the mice were monitored for 24 hours after LPS/D-GalN injection. Several liver tissues were fixed in 4% paraformaldehyde and cut into 5-μm sections, and then stained with hematoxylin-eosin (H&E). Histopathological analysis was determined with terminal deoxynucleotidyl transferase-mediated dUDP nick end labeling (TUNEL). The sections were observed in non-consecutive, free selection 400× histological fields and representative images were presented. The other liver tissues were frozen rapidly and stored at −80°C for western blot analysis.

### Determination of the levels of hepatic damage, antioxidants, and inflammatory markers

Mice blood was collected into different centrifuge tubes and centrifuged at 5000 rpm for 10 minutes, and the serums were obtained and stored at −20°C until use. ALT and AST activities were evaluated per the manufacturer's instructions (Nanjing Jiancheng Biotechnology Institute, China). The concentrations of CAT, iNOS, MDA, SOD, T-AOC, COX-2, and PGE_2_ in diverse serums were tested by use of commercial assay kits (Nanjing Jiancheng Biotechnology Institute, China).

### Western blot assay

A western blot assay was performed to observe the protein expression of the related signaling pathways and apoptosis in livers. The liver tissue lysate was separated with 12.5% SDS-PAGE and transferred onto nitrocellulose membranes. Blots were blocked in 10% skim milk at 37°C for 1.5 hours and incubated overnight at 4°C with primary antibodies directed against NF-κB p65 (1:2500), phosphor(p)-IKK (1:1000), p-IκB (1:1000), p-ERK (1:1000), p-JNK (1:1000), p-38 (1:1000), and β-actin antibody (1:5000) used as loading controls, and then incubated at room temperature for 1.5 hours with secondary antibodies of IgG-HRP-conjugated. Target proteins were visualized and quantitated by use of Image Jet software and β-actin as an internal standard.

### Immunohistochemistry staining assay

Paraffin-embedded liver sections were de-paraffinized and rehydrated, and endogenous peroxidase activity was blocked with 3% H_2_O_2_ in methanol. Antigen retrieval was performed with a 1-mM EDTA buffer (pH = 9.0) in a microwave for 3 minutes. The nonspecific protein binding was blocked by goat serum for 30 minutes. The following steps were performed per the instructions in the Histostain™ -Plus and DAB substrate kits. The sections were incubated with primary antibodies, and then continuously incubated with biotin-labeled goat antirabbit IgG-HRP. Sections were developed with 3, 3′-diaminobenzidine (DAB) solution and counterstained with hematoxylin. Images were captured with a light microscope (magnification 400×; Nikon Eclipse Ti-SR) and representative images were presented.

### Statistical analysis

All data were presented as means ± SD. Statistical significance of differences between groups was determined by one-way analysis of variance (ANOVA) performed by SPSS 18.0 software. *P* < 0.05 was considered significant.
